# Importance of the environment for gestational duration variability and correlation between relatives – results from the Medical Swedish Birth Registry, 1973-2012

**DOI:** 10.1371/journal.pone.0236494

**Published:** 2020-07-24

**Authors:** Dominika Modzelewska, Pol Sole-Navais, Ge Zhang, Louis J. Muglia, Staffan Nilsson, Bo Jacobsson

**Affiliations:** 1 Department of Obstetrics and Gynecology, Institute of Clinical Sciences, Sahlgrenska Academy, University of Gothenburg, Gothenburg, Sweden; 2 Division of Human Genetics, Department of Pediatrics, Cincinnati Children’s Hospital Medical Center, University of Cincinnati College of Medicine, Cincinnati, OH, United States of America; 3 Center for Prevention of Preterm Birth, Cincinnati Children’s Hospital Medical Center, Cincinnati, OH, United States of America; 4 Office of the President, Burroughs Wellcome Fund, Research Triangle Park, NC, United States of America; 5 Department of Mathematical Sciences, Chalmers University of Technology, Gothenburg, Sweden; 6 Department of Obstetrics and Gynecology, Sahlgrenska University Hospital, Region Västra Götaland, Gothenburg, Sweden; 7 Division of Health Data and Digitalization, Department of Genetics and Bioinformatics, Norwegian Institute of Public Health, Oslo, Norway; Hungarian Academy of Sciences, HUNGARY

## Abstract

It has been suggested that the intergenerational associations in gestational age at delivery are considerably affected by temporal changes in the environmental conditions. We explored whether changing environment affects familial resemblance of gestational age at delivery. Understanding how correlation changes in different settings allows to design better studies aimed to detect genes and environmental factors involved in the parturition process. The Swedish Medical Birth Register was used to retrieve births during 1973–2012. In total, 454,433 parent-child, 2,247,062 full sibling, 405,116 maternal half-sibling and 469,995 paternal half-sibling pairs were identified. A decreasing trend in correlation, associated with increasing age gaps, was observed among all siblings, with the largest drop for full siblings, from ρ = 0.32 (95% confidence interval (CI): 0.31, 0.33) for full siblings with one-year gap to ρ = 0.16 (95% CI: 0.10, 0.22) for full siblings with age gap above 20 years. A variation in association between full siblings born up to two years apart was observed; estimate ρ = 0.28 (95% CI: 0.26, 0.3) in 1973, and ρ = 0.36 (95% CI: 0.33, 0.38) in 2012. Observed variability in the association in gestational age at delivery between the relatives with respect to their birth year or age gap suggests the existence of temporally changing environmental factors.

## Introduction

In 2012, World Health Organization representatives called for action aimed at preterm delivery (PTD) prevention [1]. Despite continuous major public health efforts, prevalence rates remain unchanged, i.e. around 5–7% in Scandinavia [2] and around 10% in the US [3]. One difficulty in creating preventive strategies lies in a vague understanding of the biological mechanisms controlling the parturition process. Pregnancy duration is a complex phenomenon, and the onset of delivery is the result of processes engaging genetic and environmental factors. Heritability of gestational duration and preterm delivery is between 25–34% providing evidence of genetic contribution, and distinguishing maternal genetic relevance [4–9]. Genome-wide complex trait analysis provided estimate of the variance explained by all the maternal SNP at 17% for gestational duration and 23% for preterm birth liability [10]. In the genome-wide analysis, the fraction of the variance explained by the SNPs associated with gestational duration was less then 1% [10]. Among other reasons, insufficient consideration of the environment has been suggested as a possible explanation for the variation in heritability estimates [11]. While studying familial resemblance, low correlation estimates were frequently reported for parent–child cohorts. It has been suggested that the intergenerational associations are considerably affected by temporal changes in the environmental conditions [8,12]. Individuals from different generations represent widely spaced periods of time that are characterized by substantially differing environmental conditions. When it comes to pregnancy duration, differences in environment might, to a large extent, be attributed to modified obstetric practices [8]. Changes have occurred in the frequency of iatrogenic deliveries, in the prevalence of different modes of delivery, risk factors, and in gestational age estimation, all contributing to improved survival of preterm babies [8,13,14]. These changes might also have led to differing gestational ages at delivery among relatives, even if they were genetically predisposed to be born at similar gestational ages. Understanding how the correlation of gestational age at delivery between relatives changes over time might provide insights about the influence of changing environmental factors on gestational duration. Genes that have a role in the timing of normal parturition are also associated with PTD [10].

The aim of this study was to assess the presence and significance of time-related environmental (intergenerational effects) factors for the degree of association between gestational ages at delivery of relatives. For that purpose, distributions of gestational age at delivery of relatives were compared with regard to their birth years or age gaps. Next objective was to analyze the changes in the correlation estimate among relatives born in the periods of time characterized by different gestational duration distributions, and among full siblings with regard to birth years and hospital of delivery.

## Materials and methods

This study is based on a sample of pregnancies (and relevant pregnancy characteristics) reported in the Swedish Medical Birth Register (MBR [15]) during the 39-year period 1973–2012. Maintained by the Swedish National Board of Health and Welfare, the MBR contains information on 98–99% of all births in Sweden. It contains data on demography, reproductive history, as well as on complications during pregnancy, delivery and neonatal period, recorded prospectively by hospital staff [16]. Family connections between registered births were established after linking MBR to the Swedish Multi-generation Register. The cohorts of mother–child and parent–child consist the same set of children.

Data cleansing, involving removal of missing information from the best MBR estimate of gestational duration, birth year and identification number, as well as limiting the sample to live-born singleton pregnancies were the first steps before creating datasets appropriate for the analysis of parent-child, full sibling, maternal half-sibling, and paternal half-sibling pairs.

In this paper, under the term “environmental factors”, it is referred to all non-genetic factors. In order to exclude commonly acknowledged intergenerational effect (time-related environmental) factors, the full sibling sample was limited to non-iatrogenic deliveries and to neonates that survived up to 28 days after delivery. Iatrogenic delivery was defined as birth after induced onset of labor or a cesarean section before the spontaneous onset of labor (recoded as “induced” in the MBR or a reported diagnosis among the following: ICD-8 codes 651, 762.1, 762.2, 637, 661.4; ICD-9 codes 651, 762.1, 762.2, 637, 661.4; ICD-10 codes O61, O62, O60.3, O75.5). Spontaneous delivery was defined as birth after spontaneous onset of labor (recoded as “spontaneous” in the MBR or a reported diagnosis among the following: ICD-8 codes 650, 652, 657, 658, 660.2, 660.3, 660.4, 661.1; ICD-9 codes 650, 658, 652, 657, 660.2, 660.3, 660.4, 661.1; ICD-10 codes O81, O84.0, O84.1, O75.6, O60.1, O60.2, O80, O42). Births with unclassified mode of delivery were excluded from analysis.

Distributions of gestational age at delivery among relatives were compared by Q-Q plots in order to detect whether environmental conditions change. Associations between relatives were obtained from correlation analysis. Differences in the correlations between relatives born in different periods of time might be due to differences in the age of their mothers during pregnancy and her parity. In order to assess the existence of other time-related environmental factors, gestational age at delivery duration was adjusted for the maternal age at delivery, parity. The adjustment consisted of two steps, estimating the effect of maternal age and parity on gestational duration, and adjusting gestational duration in a way as if all births were to a woman of age between 20–30 years old and of parity two. More about gestational duration adjustment are found in S1 Table. In additional analyses for siblings, gestational age at delivery was also adjusted for maternal body mass index (BMI). Since the results between correlation estimates non- and adjusted didn’t differ, in the paper, there are provided results based on adjusted gestational duration. In order to assure that the results are not affected by temporal changes in the estimation method of gestational duration, we performed additional sensitivity analysis using gestational durations estimated solely on last menstrual period (LMP). Statistical significance of a change in the correlation was assessed based on the significance of interaction term (gestational age at delivery and birth year, alpha < 0.05). Additionally, logistic regression was performed for the pairs of parents and their first child, full- and half-siblings. Siblings’ samples were restricted to the children of the first and second parity. For all the types of relatives, regression models were adjusted for maternal age at delivery. Additional adjustment for maternal BMI was made for siblings. All analyses were performed in R software, version 3.5.1.

For the analyses of relatives born in different periods of time, the distinct birth year periods were determined based on the alterations that occurred in the population gestational duration distribution. In this paper, distinct (minimum 10 years difference) periods of birth years were called closely and widely spaced accordingly to the degree to which population gestational duration distribution differed (for more information, see S1 Fig). The study was approved by Regional Ethic Committee of the Western Health Care Region in Sweden (Dnr. 576–13). Since MBR is a national population-wide database, no informed consent is required. Individual-level data are anonymous. Personal identification numbers are kept and known only to the National Board of Health and Welfare.

## Results

### Cohort

**Fig 1:**
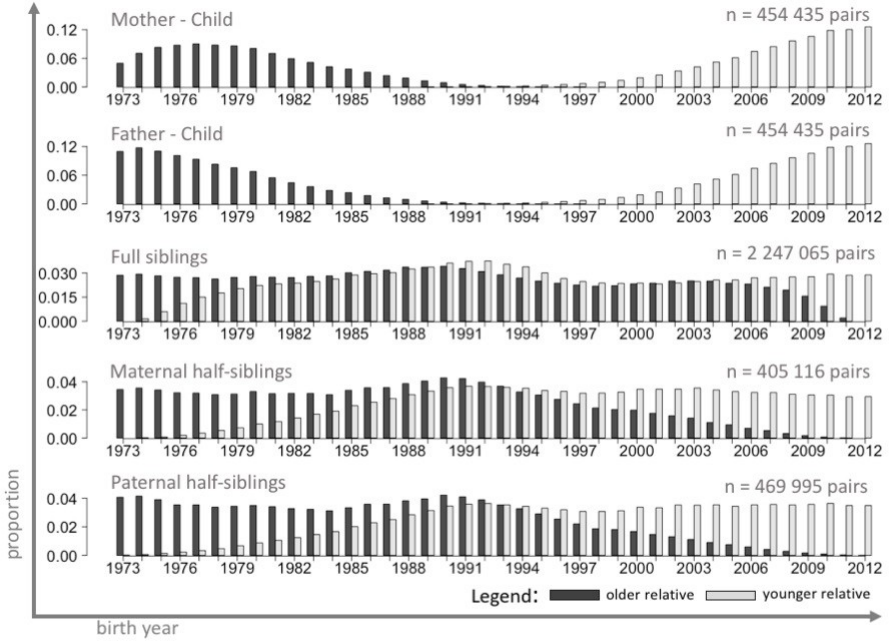
Distributions of birth years in cohorts of paired relatives, Swedish Medical Birth Register 1973–2012. The figure shows the distributions of birth years in cohorts of paired relatives. The cohorts of relatives were created so that one cohort consists of individuals (dark grey bars) and the second consists, matched to those individuals, of family members born later (light grey bars). Based on a sample of live-born singletons, no other exclusion criteria, n = 3,914,795.

**Fig 2:**
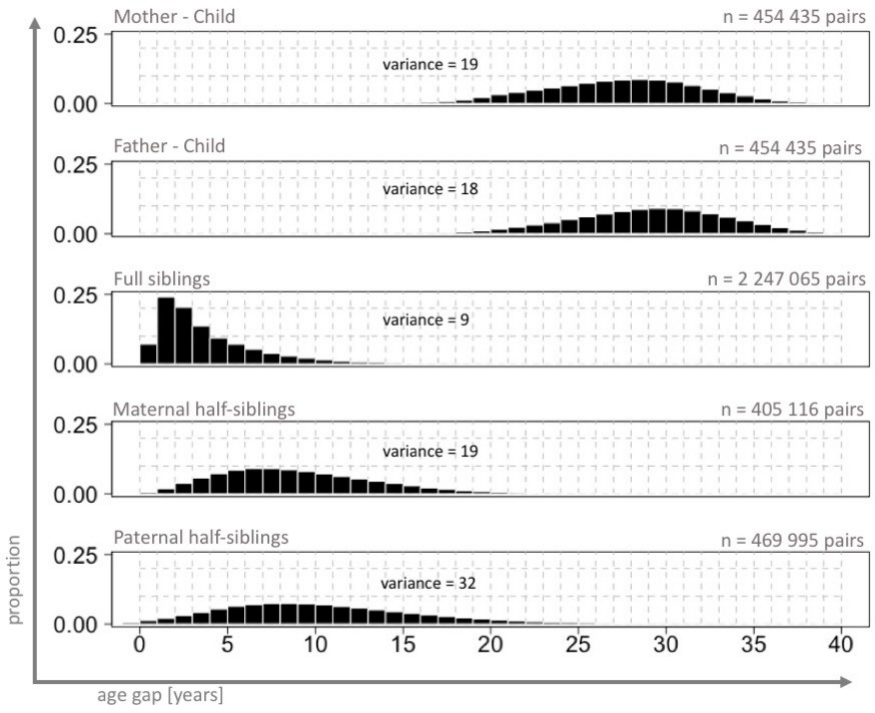
Distributions of age gaps in cohorts of paired relatives, Swedish Medical Birth Register 1973–2012. The figure shows the distributions of the differences between the birth years of the relatives (from 0 up to 40 years difference). Based on a sample of live-born singletons, no other exclusion criteria, n = 3,914,795.

The initial dataset consisted of 4,079,106 deliveries. Quality control and cleansing of the dataset resulted in removal of 164,311 deliveries, so that the main database remaining for analysis consisted of 3,914,795 entries. This resulted in 454,433 parent-child pairs, 2,247,062 full sibling pairs, 405,116 maternal half-sibling pairs, and 469,995 paternal half-sibling pairs. Cohorts of relatives represented distinct (parents-offspring pairs) or overlapping (sibling pairs) birth years (Fig 1). All family member pairs except full siblings were characterized by flat age gap distributions, yielding a broad range of possible birth year differences (Fig 2). Parent-offspring pairs were characterized by large gaps between their birth years (quartiles: 25, 28, 31 years and 27, 30, 33 years for mother- and father-offspring pairs, respectively). Relatively large age gaps were also observed among maternal and paternal half-siblings (quartiles: 6, 9, 12 years and 7, 10, 14 years, respectively). Around five percent of full siblings were born more than 10 years apart.

### Detection of time-related distribution differences

**Fig 3:**
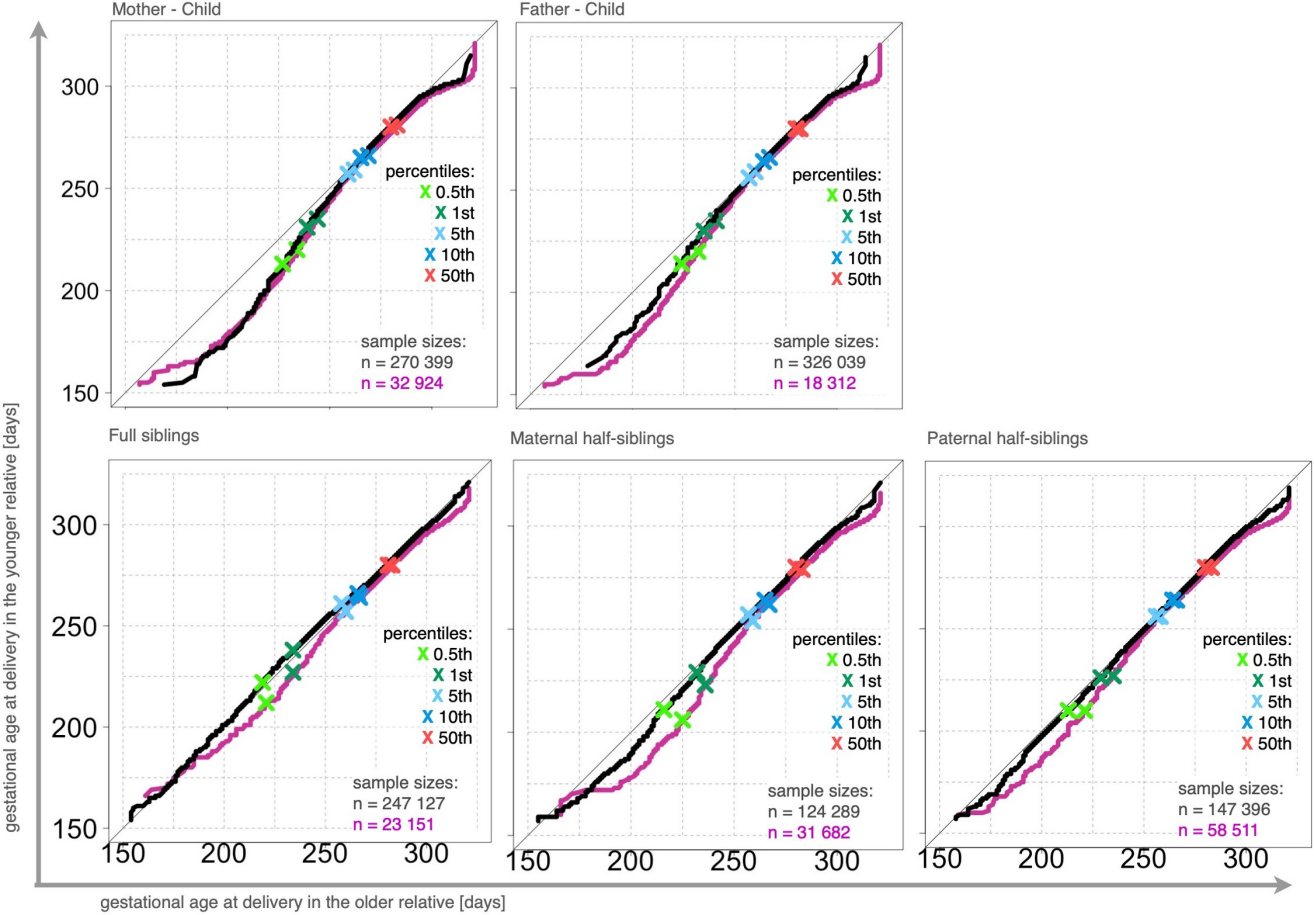
Q-Q plots comparing gestational age distributions among relatives, Swedish Medical Birth Register 1973–2012. The figure shows the differences in the shape of the distribution of gestational ages at delivery in matched relatives. Two Q-Q plot lines are shown. The pink line represents the cohorts of relatives born in the periods characterized by more different gestational duration distributions (called widely spaced periods), and the black line represents the cohorts of relatives born in the periods characterized by less different gestational duration distributions (called closely spaced periods). For the selection of birth year periods, see S1 Fig. Q-Q plots generated from a sample of live-born singletons, no other exclusion criteria, n = 3,914,795. The x-axis represents gestational age in the older relatives, and the y-axis gestational age in the younger relatives.

Distribution differences were detected among all types of family member pairs (Fig 3). In the Q-Q plots, the curves are below the diagonal line indicating that the distributions of all relatives born in later periods contain more preterm deliveries compared to the distributions among family members born in earlier periods (McNemar test, p < 0.001, among all family member pairs). Similar QQ-plots were obtained when the analyses were based on gestational durations estimated solely on LMP information. As expected, larger distribution differences were observed when individuals born in periods that were farther apart were matched into pairs, except in the case of mother-child (concerning the selection of birth year periods, see S1 Fig). The lack of differences between the two sub-cohorts (curves) in the Q-Q plots in mother-child pairs was possibly due to differences in maternal characteristics that cancels the gap effect. In the mother-child cohort, the mothers in the sample of relative pairs from more closely spaced periods were younger (median age: 23) than those (median age: 30) in the pairs from more widely spaced periods.

### Correlation estimates with respect to birth year periods and age gaps

**Fig 4:**
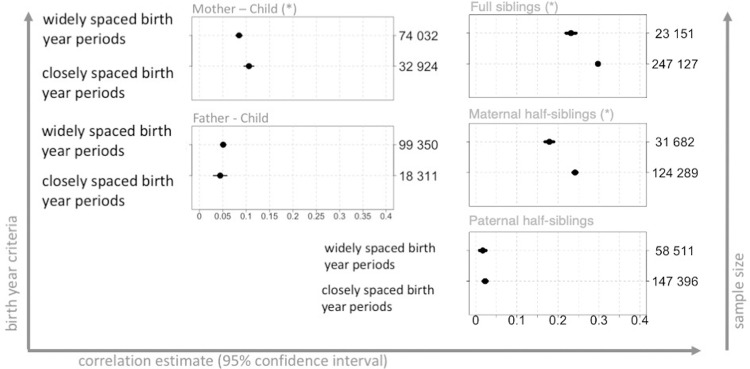
Correlation estimates for gestational age in relatives with regard to birth year periods, Swedish Medical Birth Register 1973–2012. The figure shows the correlation estimates (black dots), with 95% confidence intervals (horizontal bars), for different types of relatives, with regard to birth year periods. Gestational duration was adjusted for maternal age at delivery and parity (for details, see S1 Table). Statistically significant change in the correlation estimates was depicted by a star next to family type headline. Analysis performed on samples limited to live births, no other exclusion criteria, n = 3,914,795. The left-hand y-axis indicates the birth year criteria used for extraction of the pairs of relatives, for details, see S1 Fig. The right-hand y-axis depicts the sample sizes and the x-axis shows the estimate size. Note that, due to large dot size (for visibility purposes), small confidence intervals may not be visible.

Correlation estimates for closely and widely spaced periods differed significantly in the samples of full siblings (p < 0.001, for interaction term), maternal half-siblings (p < 0.001, for interaction term), and mother-child pairs (p = 0.002, for interaction term). The strongest change in the association magnitude was in the samples of full siblings and maternal half-siblings, from ρ = 0.23 (95% CI: 0.22, 0.24) to ρ = 0.3 (95% CI: 0.29, 0.30) and from ρ = 0.18 (95% CI: 0.17, 0.19) to ρ = 0.24 (95% CI: 0.24, 0.25), respectively (Fig 4), (For the selection of birth year periods, see S1 Fig). Mother-child correlation increased from 0.09 (95% CI: 0.08, 0.09) to 0.11 (95% CI: 0.09, 0.12).

**Fig 5:**
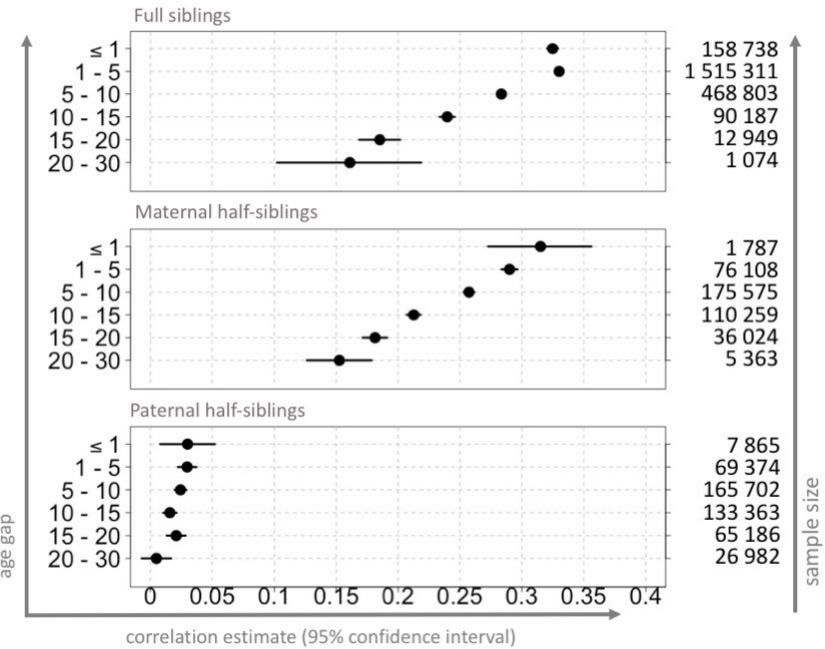
Correlation estimates for gestational ages among relatives with regard to age gaps, Swedish Medical Birth Register 1973–2012. The figure shows the correlation estimates (dots), with 95% confidence intervals (horizontal bars) for sibling pairs, with regard to the difference in birth years. Gestational duration was adjusted for maternal age at delivery and parity (for details, see S1 Table). Analyses performed on samples limited to live births, no other exclusion criteria, n = 3,914,795. The left-hand y-axis indicates the range of age gap criteria used for extraction of the siblings. The right-hand y-axis depicts the sample sizes, and the x-axis shows the correlation values. Note that, due to large dot size (for visibility purposes), small confidence intervals may not be visible.

Significantly decreasing trend in correlation estimates, associated with increasing age gaps, was observed among all siblings (Fig 5 and S1 Appendix). This pattern was only broken in the case of full sibling pairs born up to one year apart, for which there was a relatively lower regression estimate. Correlation estimates dropped from ρ = 0.32 (95% CI: 0.31, 0.33) to ρ = 0.16 (95% CI: 0.10, 0.22) for full siblings; from ρ = 0.31 (95% CI: 0.27, 0.35) to ρ = 0.15 (95% CI: 0.13, 0.18) for maternal half-siblings; and from ρ = 0.03 (95% CI: 0.01, 0.05) to ρ = 0.01 (95% CI: -0.01, 0.02) for paternal half-siblings. Changes in the correlation coefficient followed the same pattern in the analyses performed on gestational durations estimated solely on LMP information.

### Distribution differences among spontaneously delivered neonatal period survivors

**Fig 6:**
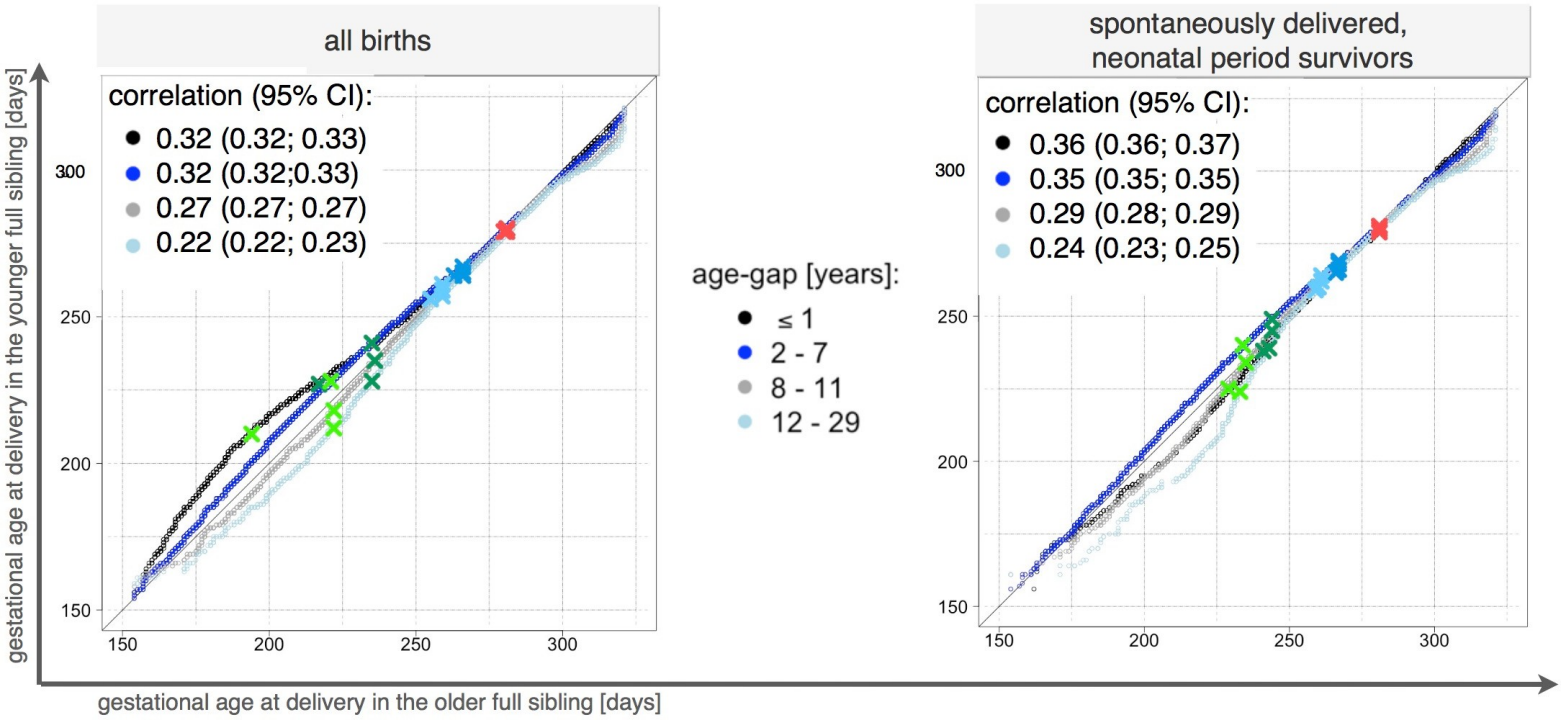
Q-Q plots comparing the gestational age distributions of full siblings, during the period 1973–2012, Swedish Medical Birth Register 1973–2012. The figure shows the differences in the distribution of gestational duration in full siblings with regard to the age gap between them. The x-axis represents the value of gestational age in the older full siblings, and the y-axis represents that in the younger full siblings. Q-Q plots were performed on two types of samples. The graph on the left depicts the Q-Q plots based on the samples restricted to live births. The graph on the right depicts the Q-Q plots performed on a sample of spontaneously delivered neonatal-period (28 days) survivors, n = 1,591,769. The correlation estimates are depicted on the graphs.

Differences in distributions were observed among full siblings, regardless the age gap between them. In the QQ-plot for full siblings born at most seven years apart, the curve was above the diagonal line indicating that more PTD children were in the cohort of older sibling (Fig 6 left panel). Opposite pattern was for full siblings born eight and more years apart. In the QQ-plot, the curve was below the diagonal line indicating more PTDs in the cohort of younger child (Fig 6 left panel). The pattern of distribution differences changed for full siblings born up to 1 years apart when the sample was restricted to spontaneously delivered children who survived the neonatal period (28 days). In the QQ-plot, the curve was below the diagonal line indicating more PTDs in the cohort of younger sibling (Fig 6 right panel). A general pattern of decreasing estimate for correlation was observed among the full siblings as the age differences increase. Exclusion of iatrogenic deliveries and neonatal deaths led to increased correlation estimates among full siblings born up to 7 years apart.

### Correlation between full siblings, born up to two years apart, over the years

**Fig 7:**
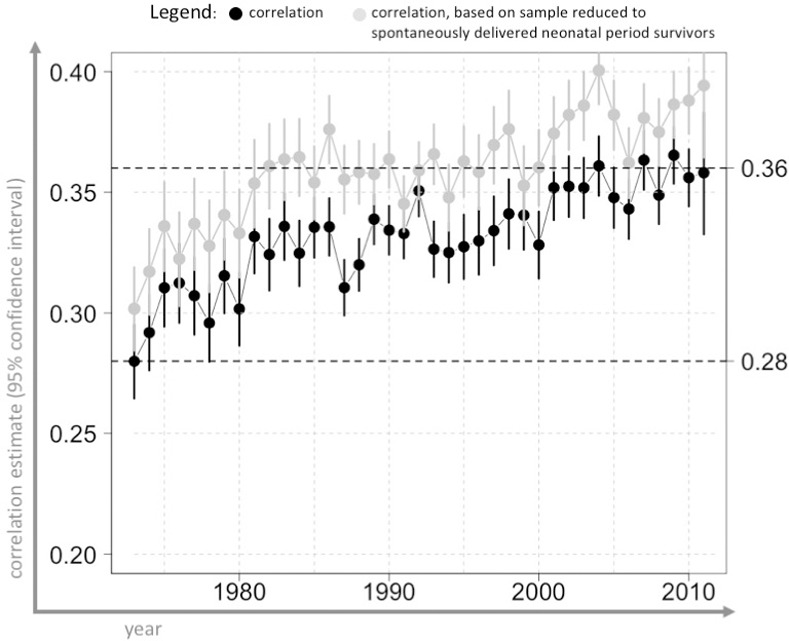
Correlation estimates for gestational duration in full siblings born up to two years apart in 1973–2012, Swedish Medical Birth Register 1973–2012. The figure shows the correlation estimates (black dots), with 95% confidence intervals (horizontal bars), for siblings born up to two years apart over the years. Gestational duration was adjusted for maternal age at delivery and parity (for details, see S1 Table). Analysis performed on samples restricted to live births, no other exclusion criteria, n = 2,247,062 pairs. Grey dots depict the estimates obtained from the analysis run on the sample restricted to spontaneously delivered neonatal-period (28 days) survivors, n = 1,591,769 pairs. The x-axis indicates the year of birth of the older full sibling.

**Fig 8:**
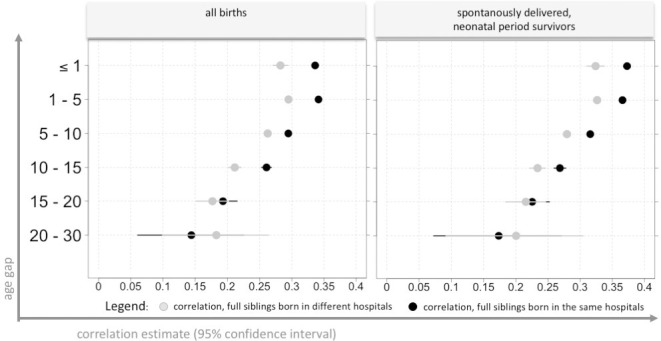
Correlation estimates for gestational duration in full siblings, with regard to the age gap between them and to the hospital at which the mother gave birth, Swedish Medical Birth Register 1973–2012. The figure shows the correlation estimates (dots), with 95% confidence intervals (horizontal bars), for full siblings, with regard to the difference between birth years, and the hospital at which the mother gave birth. Gestational duration was adjusted for maternal age at delivery and parity (for details, see S1 Table). The graph on the left represents the estimates of correlation performed on the sample restricted to live births, while the graph on the right serves to depict the correlation estimates performed on a sample restricted to spontaneously delivered neonatal-period (28 days) survivors. Different-colored dots mark whether full siblings were born in the same hospital (black–same hospital; grey–different hospital). The y-axis indicates the age gap criteria used for the extraction of the siblings. The x-axis shows the association estimate values.

Variation in the correlation estimates was observed among full siblings born up to two years apart (Fig 7 and S1 Appendix); the correlation magnitude depended on during which period the individuals were born. Correlation estimates varied significantly over the years, from ρ = 0.28 (95% CI: 0.26, 0.3) in 1973 to ρ = 0.36 (95% CI: 0.33, 0.38) in 2012. Larger, but similarly varying, correlation estimate was observed when estimation was performed on a sample of full siblings restricted to spontaneously delivered survivors of the neonatal period. It has been observed, among full siblings born up to ~15 years apart, that the association was significantly lower when they were born in different hospitals (Fig 8 and S1 Appendix). Changes in the correlation coefficient followed the same pattern in the analyses performed on gestational durations estimated solely on LMP information.

#### Relative risk for preterm delivery

Women had increased risk to deliver first child preterm if they were born preterm themselves (odds ratio (OR) = 1.41, 95%CI: 1.31, 1.51, n = 256,868). A small increase was also found for the fathers and their first child (OR = 1.1, 95%CI: 1.02, 1.19, n = 256,868). When restricting the sample to siblings of the first and second parity, there is an increased risk for a child to be born preterm if his/her full- or maternal half-sibling was born preterm as well (respectively: OR = 6.9, 95%CI: 6.7, 7.2, n = 721,694; OR = 4.9, 95%CI: 4.35, 5.4, n = 74,633). No increase was found for paternal half-siblings (OR = 0.92, 95%CI: 0.79, 1.08, n = 65,835).

## Discussion

The main finding of this study is variability in the correlation between gestational ages at delivery in relatives, after stratification by their birth years. The association between maternal and child’s gestational age at delivery is significantly lower if there are large gaps in their ages, even after adjustment for maternal age at delivery and parity. Similarly, the same significantly decreasing trend in the degree of association with increasing age gap was observed among siblings. Variability in the correlations over the years was also observed among full siblings born up to two years apart. This heterogeneity of associations was observed both when it came to correlations based on unadjusted gestational age and adjusted for maternal age and parity. The extent to which correlations varied also depended on the extent to which the distributions of the cohorts of relatives differed. The pattern of larger distribution differences and larger decreases in the degree of association was observed among mother-child, but not father-child, pairs. It is possible that the low correlation estimates among the father-child pairs prevented detection of this variation. Similarly, in the case of siblings, the association decreased with age gap among those with relatively high overall correlations, e.g. full siblings and maternal half-siblings, but not paternal half-siblings. Our finding of a lower correlation among paternal half-siblings compared with full siblings or maternal half-siblings once again suggested that the genetic control of gestational duration comes mainly from the maternal side [5,6,9]. In addition, the suggestion that heritable factors for preterm delivery work through the mother is supported by our findings of a larger risk ratio on the maternal side. Other studies based on the Swedish MBR provide similar conclusions [17,18].

Different researchers have expressed various opinions about the effect of time-related environmental factors on the estimates of association between the relatives as well as on heritability. While some think that time-related environmental factors have no effect on the association estimates [13], others [20] draw the same conclusions as those presented in this paper, i.e. a diminished degree of association due to time-related environmental factors. Wu et al. discussing the significance of the intergenerational effect in terms of heritability analysis, suggested that the pattern of difference in the distributions among parents and their children leads to non-linear associations between parents and offspring, which might bias the heritability estimate.

While there seems to be considerable interest in understanding the composition of observed parent-offspring associations, there is, to our knowledge, no previous study analyzing these associations with regard to birth years of relatives. Some [4,8,18–22], but not all [17,23–27]; studies on intergenerational associations address, to varying extents, the problem related to intergenerational effect factors. However, when exploring biologically determined phenotypic similarity between relatives, time-related environmental factors might be considered methodologically for another reason than that of managing the effect of the artificially diminished degrees of similarity attributed to those factors, as in some studies [4,20]. Taking those factors into account in methodology might also be motivated by the aim of evaluating pregnancy free from pathological conditions [4,9]. In that case, when addressing the heterogeneous collection of factors affecting pregnancy duration, methodology is instead driven by taking the influence of biological rather than external environmental factors on the association estimates into account. Taking time-related factors, from the perspective of biological rather than environmental factors, into account in methodology might be enhanced by accepting the assumption that differences in distributions only exist among intergenerational cohorts. As it has been suggested [8,20], distribution differences between cohorts from different generations are caused mainly by changes in obstetric practices which tend to artificially shorten the span of pregnancy. However, this study found that the distribution differences were not solely features of intergenerational cohorts, but also existed among full siblings with small age gaps. Due to the opposite directions in the respective shifts in distributions among full siblings with short and large age gaps, this phenomenon has been overlooked by other researchers [8] and the differences mistakenly interpreted as an exclusive feature of intergenerational cohorts. The comparable sizes of the distribution differences in the parent-offspring and closely aged full sibling pairs add to the paradigm of a large range of factors affecting pregnancy duration. However, while there is a similarity in distribution differences between different types of relatives, the correlation estimates differ; they are small among parent-offspring pairs and relatively large among full sibling pairs. Larger correlation estimates between full siblings in comparison to correlation estimates between parent-offspring pairs, even in the presence of distribution differences, might be explained by the existence of more factors contributing to the similarity between full siblings than parent-offspring, such as dominance variance or shared environment, both commonly mentioned. This study shows that the associations between full siblings born up to two years apart varies over the years. This suggests variability in the contributions of external factors to the degree of association between relatives. The hypothesis of varying external environmental conditions is supported by the variability in the associations between full siblings with regard to the hospital at which they were born, as reported in this study. Differences in healthcare services might add to variability in the population and affect the degree of resemblance between relatives. Varying healthcare practices in Sweden was suggested by Murray et al. [28] as one of the explanations for observed differences in PTD rates across the country. This diversity in healthcare practices, among other factors of a clinical nature, includes disagreement on normal pregnancy duration. Due to the five-week window (37^th^ to 42^nd^ week of gestation) in how normal pregnancy duration is defined [29], there is no consensus. And so, across time and countries, and within countries, different values between 279 and 282 days have been selected [30] to represent the full-term pregnancy norm. If the corresponding expected pregnancy duration is not taken into account, estimates of gestational duration based on due-date might contribute to differences between relatives, especially when dichotomized variable preterm/term is used for comparison purposes.

While analyzing the variability of the association between relatives, the best estimate of gestational duration provided by the MBR was used. The MBR best estimate selection is based on assumptions about the accuracy of various methods [15]. Since the available estimates were based on different methods, the best estimate of the MBR was heterogeneous. Some researchers [31] have reported that introduction of ultrasound-based gestational duration estimation was associated with changes in pregnancy duration distribution. The increasing trend towards ultrasound-based estimation, reaching more than 80% prevalence after 1995, contributed to the distribution difference between relatives. Relatives born in periods characterized by a mixture of methods for gestational duration estimation might exhibit lower similarity than relatives born in periods during which one method prevailed. However, the pattern of decreasing correlation over time and along with increasing age gap was observed regardless the estimation method was taken into account. Sensitivity analyses confirmed that there are many environmental changes, not only limited to the technical factors of gestational age estimation methods.

Due to poor reporting on the mode of onset of delivery during certain periods in the MBR, the determination of the onset of delivery (spontaneous or iatrogenic) might be subject to misclassification. Mode of onset of delivery began to be reported in the MBR in 1990. Therefore, in this study, onset of delivery before 1990 was assessed based exclusively on ICD codes. Additionally, in the MBR, the information required to calculate maternal BMI is consistently available only from 1992 what rules out parent-offspring and limits the analyses to sibling pairs.

## Conclusions

Observed variability in the association in gestational age at delivery between the relatives with respect to their birth year or age gap suggests the existence of temporally changing environmental factors.

## Supporting information

S1 Table Expected change in the gestational duration given maternal age at delivery and parity. Table presents estimates and 95% confidence intervals (CI) for maternal age category, and parity. The reference group were 20–30 years for maternal age (n = 2,255,865 births) and two for parity (n = 1,448,982 births). Gestational duration, reported in Swedish Medical Birth Register, was adjusted accordingly to the expected shift from the reference population, i.e. women giving birth at age between 20 and 30 years old and of parity two.
(DOCX)

S1 Fig Birth year ranges in the cohorts of paired relatives. Birth-years (black or pink rectangles) in the cohorts of paired relatives were selected based on gestational duration distribution in the population (n = 3,914,795). Nine percentiles (0.5th, 1st, 2.5th, 10th, 30th, 50th, 70th and 90^th^) of gestational duration distribution were plotted over the years 1973–2012 (black, solid, horizontal lines). Based on the visual assessment of gestational duration distribution, the periods of birth years in the cohorts of parents and their children were selected so that relatives were born in the periods of time characterized by more (black rectangle) and less (pink rectangle) similar distribution. Same was done for all sibling pairs. The upper part of a graph shows birth year periods in the cohort of parents and their offspring, the lower part of a graph shows birth year periods in the cohort of siblings. In the paper, cohorts of relatives born in the periods of time characterized by less or more similar gestational duration distributions were called as relatives matched from widely or closely spaced periods of time.
(TIFF)

S1 Appendix Gestational duration adjustment for maternal BMI. Appendix includes results of correlation analyses performed on gestational duration adjusted for maternal body mass index.
(PDF)
