# Supplementary material for: Importance of the environment for gestational duration variability and correlation between relatives – results from the Medical Swedish Birth Registry, 1973-2012
Source: PLoS One. 2020 Jul 24;15(7):e0236494. doi: 10.1371/journal.pone.0236494 (PMC7380618; doi:10.1371/journal.pone.0236494)
Supplement: S1 Table — Table presents estimates and 95% confidence intervals (CI) for maternal age category, and parity. The reference group were 20–30 years for maternal age (n = 2,255,865 births) and two for parity (n = 1,448,982 births). Gestational duration, reported in Swedish Medical Birth Register, was adjusted accordingly to the expected shift from the reference population, i.e. women giving birth at age between 20 and 30 years old and of parity two. (DOCX) [file pone.0236494.s001.docx]

| variable | category | beta | 95% confidence interval | n |
| --- | --- | --- | --- | --- |
| parity | **1** | -0.12 | -0.15; -0.09 | 1 665 398 |
|  | **3** | -0.20 | -0.24; -0.16 | 570 854 |
|  | **4** | -1.00 | -1.07; -0.94 | 157 742 |
|  | **5** | -1.50 | -1.62; -1.38 | 45 350 |
|  | **6** | -1.27 | -1.47; -1.05 | 15 198 |
|  | **7** | -0.72 | -1.04; -0.40 | 5 979 |
|  | **8+** | -0.51 | -0.86; -0.16 | 5 133 |
| maternal age | **12-20** | -0.42 | -0.49; -0.36 | 164 544 |
|  | **30-35** | -0.35 | -0.38; -0.32 | 1 031 529 |
|  | **35-40** | -1.20 | -1.25; -1.16 | 396 683 |
|  | **40-57** | -2.57 | -2.67; -2.47 | 66 174 |
