# Supplementary figures and images for: Importance of the environment for gestational duration variability and correlation between relatives – results from the Medical Swedish Birth Registry, 1973-2012
Source: PLoS One. 2020 Jul 24;15(7):e0236494. doi: 10.1371/journal.pone.0236494 (PMC7380618; doi:10.1371/journal.pone.0236494)

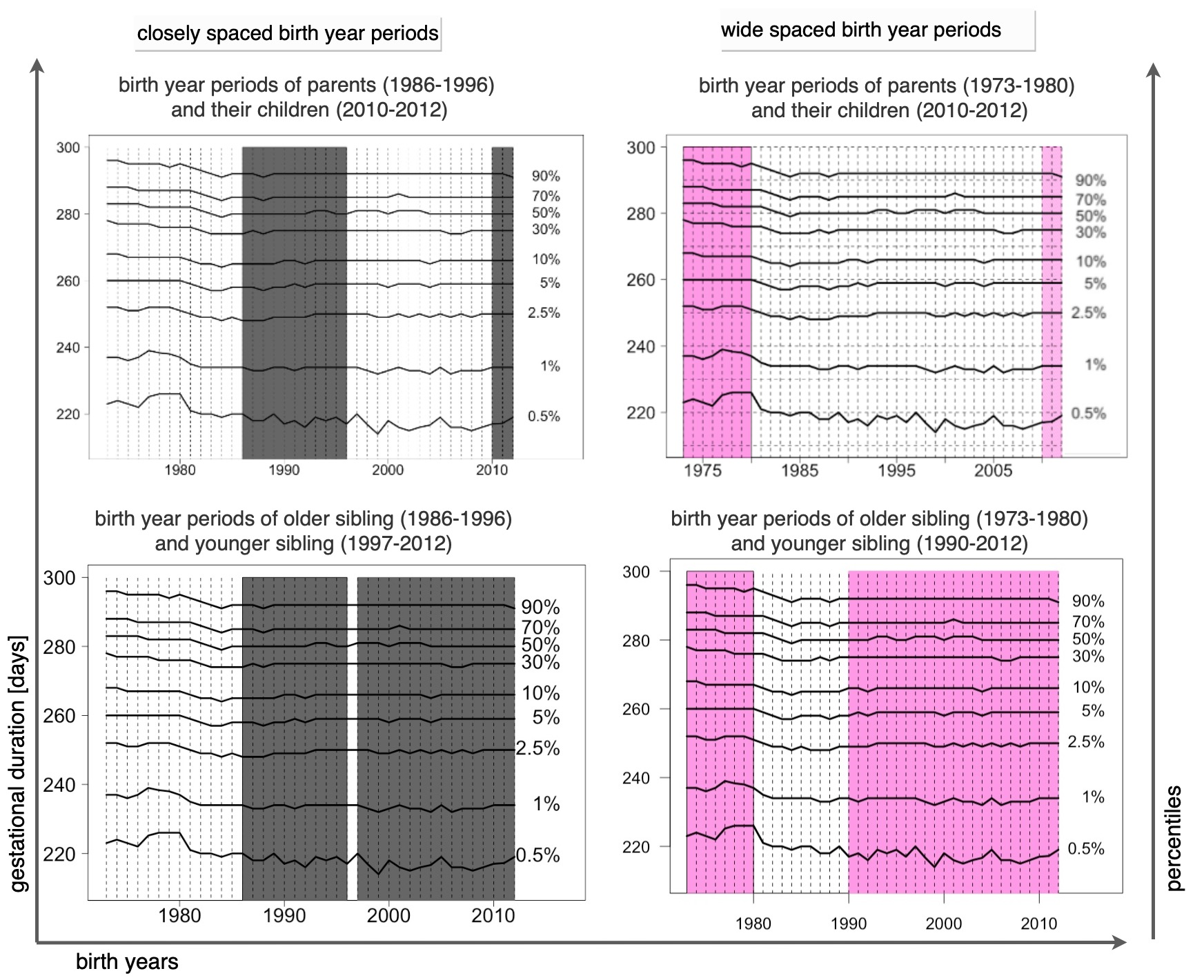

Supplement: S1 Fig — Birth-years (black or pink rectangles) in the cohorts of paired relatives were selected based on gestational duration distribution in the population (n = 3,914,795). Nine percentiles (0.5th, 1st, 2.5th, 10th, 30th, 50th, 70th and 90th) of gestational duration distribution were plotted over the years 1973–2012 (black, solid, horizontal lines). Based on the visual assessment of gestational duration distribution, the periods of birth years in the cohorts of parents and their children were selected so that relatives were born in the periods of time characterized by more (black rectangle) and less (pink rectangle) similar distribution. Same was done for all sibling pairs. The upper part of a graph shows birth year periods in the cohort of parents and their offspring, the lower part of a graph shows birth year periods in the cohort of siblings. In the paper, cohorts of relatives born in the periods of time characterized by less or more similar gestational duration distributions were called as relatives matched from widely or closely spaced periods of time. (TIFF) [file pone.0236494.s002.tiff]
