# Supplementary material for: Importance of the environment for gestational duration variability and correlation between relatives – results from the Medical Swedish Birth Registry, 1973-2012
Source: PLoS One. 2020 Jul 24;15(7):e0236494. doi: 10.1371/journal.pone.0236494 (PMC7380618; doi:10.1371/journal.pone.0236494)
Supplement: S1 Appendix — Appendix includes results of correlation analyses performed on gestational duration adjusted for maternal body mass index. (PDF) [file pone.0236494.s003.pdf]

Gestational duration adjustment for maternal body mass index (BMI)

Having crude linear regression model with gestational duration coded in days, and maternal BMI reference group between 18.5 and 24, the association estimates are as follow: β = -2.13 for BMI < 18.5, β = 0.43 for 25 ≤ BMI < 30, and β = -0.09 for BMI ≥ 30, all statistically significant (p-value < 0.01). Gestational age at delivery was adjusted accordingly to the expected shift from the reference group.

Results

Presented below Figures 1-3 correspond to the main Figures 5, 7 and 8. Figures 1-3, in contrast to the Figures 5, 7-8, present correlation estimates based on relatives' gestational ages at delivery adjusted for the maternal BMI.

Fig 1 (corresponding to the main Fig 5). Correlation estimates for gestational ages among relatives with regard to age gaps, Swedish Medical Birth Register 1973-2012.

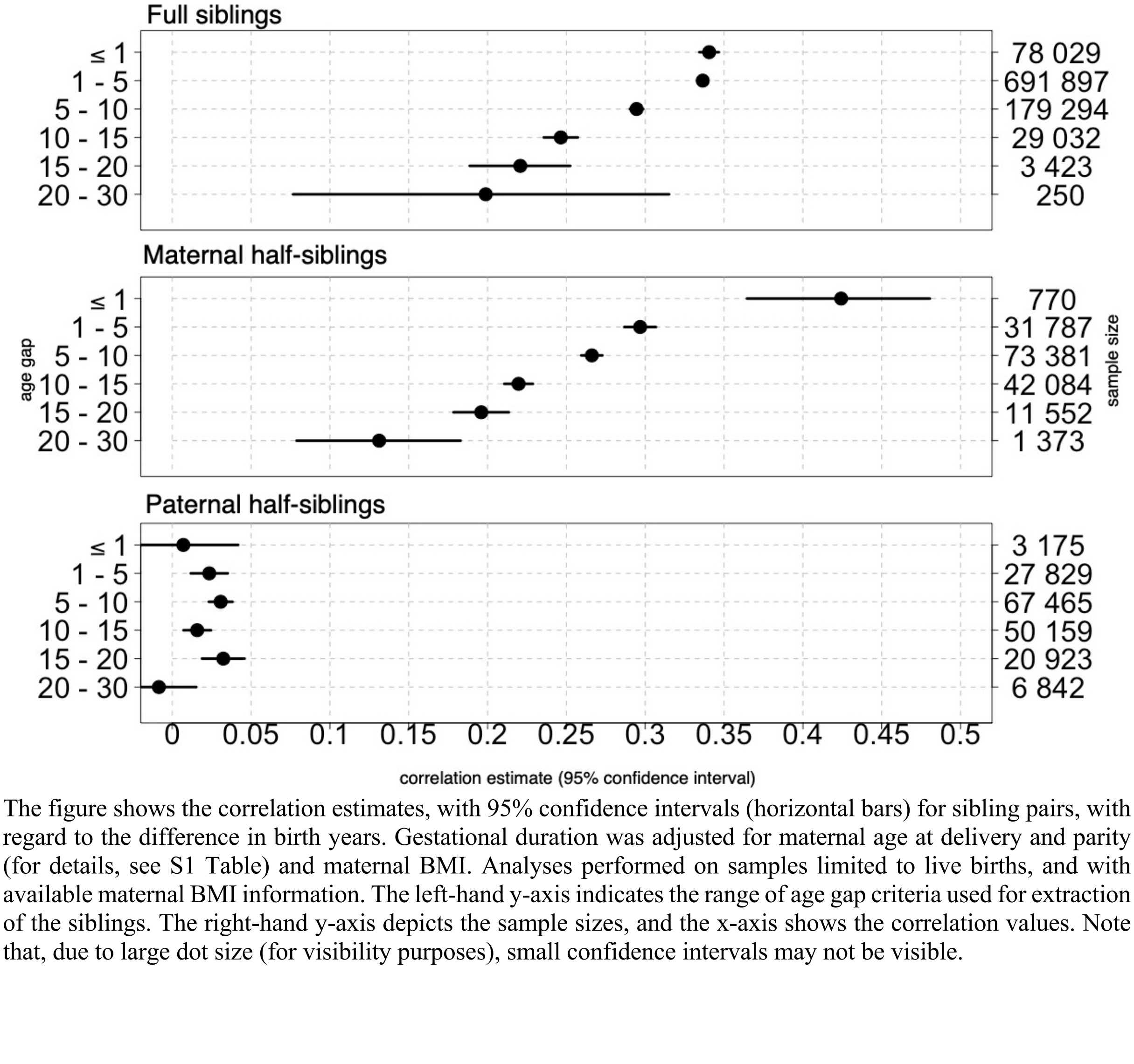

The figure shows the correlation estimates, with 95% confidence intervals (horizontal bars) for sibling pairs, with regard to the difference in birth years. Gestational duration was adjusted for maternal age at delivery and parity (for details, see S1 Table) and maternal BMI. Analyses performed on samples limited to live births, and with available maternal BMI information. The left-hand y-axis indicates the range of age gap criteria used for extraction of the siblings. The right-hand y-axis depicts the sample sizes, and the x-axis shows the correlation values. Note that, due to large dot size (for visibility purposes), small confidence intervals may not be visible.

Fig 2 (corresponding to the main Fig 7). Correlation estimates for gestational duration in full siblings born up to two years apart in 1973-2012, Swedish Medical Birth Register 1973-2012.

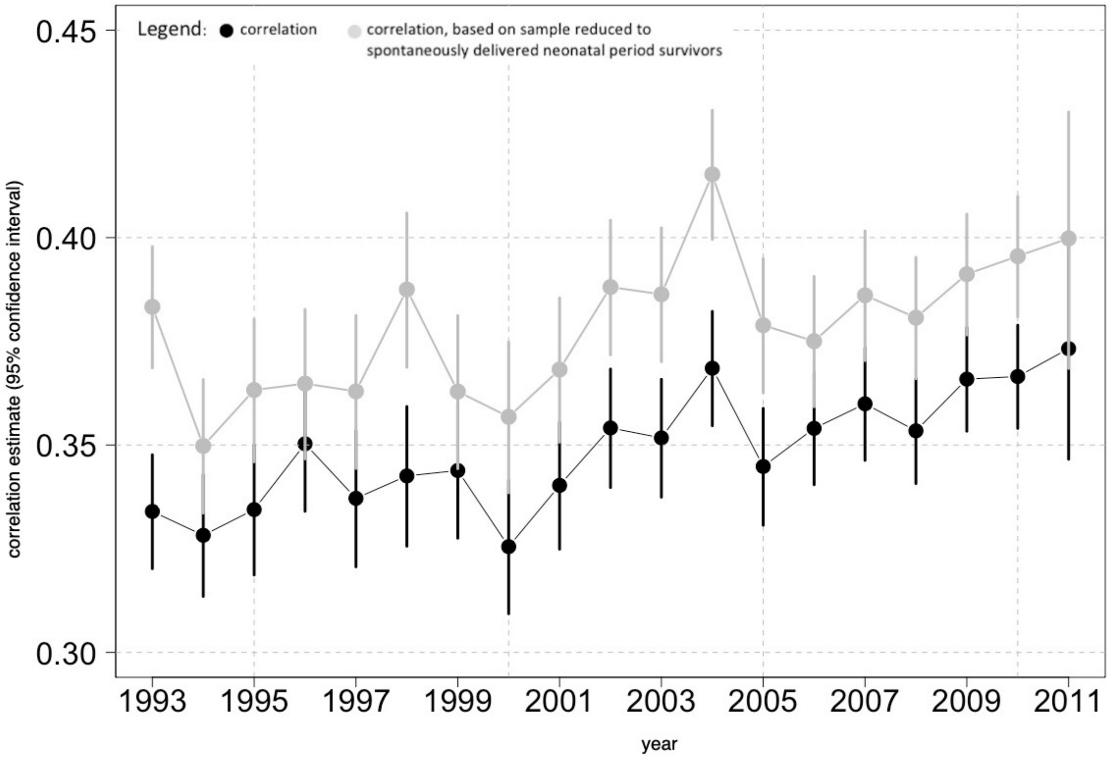

The figure shows the correlation estimates (black dots), with 95% confidence intervals (horizontal bars), for siblings born up to two years apart over the years. Gestational duration was adjusted for maternal age at delivery and parity (for details, see S1 Table) and maternal BMI. Analysis performed on samples restricted to live births, and with available maternal BMI information. Grey dots depict the estimates obtained from the analysis run on the sample restricted to spontaneously delivered neonatal-period (28 days) survivors. The x-axis indicates the year of birth of the older full sibling.

Fig 3 (corresponding to the main Fig 8). Correlation estimates for gestational duration in full siblings, with regard to the age gap between them and to the hospital at which the mother gave birth, Swedish Medical Birth Register 1973-2012.

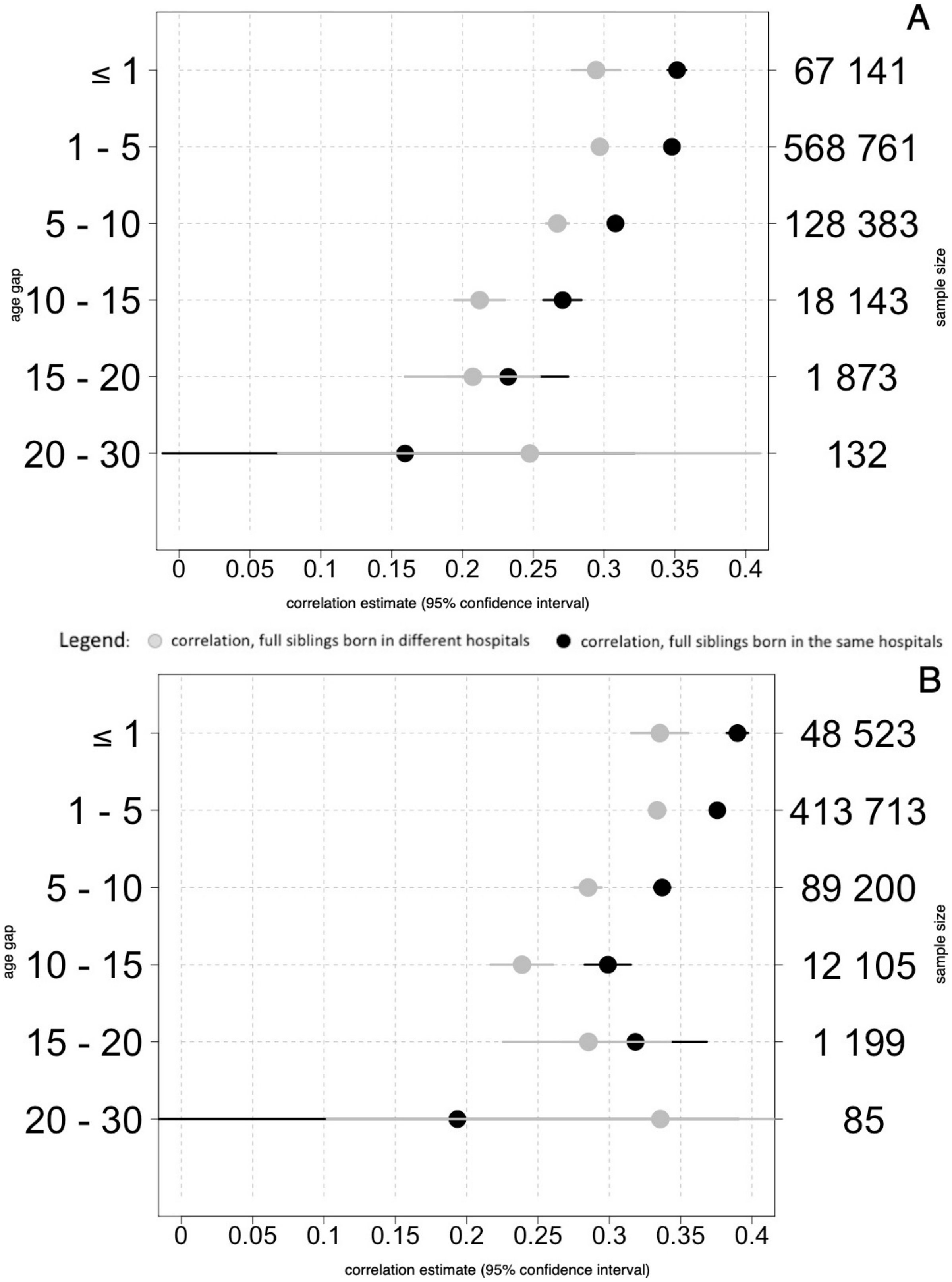

The figure shows the correlation estimates (dots), with 95% confidence intervals (horizontal bars), for full siblings, with regard to the difference between birth years, and the hospital at which the mother gave birth. Gestational duration was adjusted for maternal age at delivery and parity (for details, see S1 Table) and maternal BMI. The

upper graph (A) represents the estimates of correlation performed on the sample restricted to live births, while the bottom graph (B) serves to depict the correlation estimates performed on a sample restricted to spontaneously delivered neonatal-period (28 days) survivors. Different-colored dots mark whether full siblings were born in the same hospital (black – same hospital; grey – different hospital). The left-hand y-axis indicates the age gap criteria used for the extraction of the siblings. The right-hand y-axis depicts the sample sizes. The x-axis shows the association estimate values.
